# Endoscopic retrograde cholangiopancreatography in children with pediatric congenital biliary dilatation associated with pancreatobiliary maljunction: experience from a tertiary center

**DOI:** 10.3389/fped.2024.1484375

**Published:** 2025-01-06

**Authors:** Giovanni Rollo, Valerio Balassone, Simona Faraci, Filippo Torroni, Luigi Dall'Oglio, Paola De Angelis, Tamara Caldaro

**Affiliations:** ^1^ Digestive Endoscopy and Surgery Unit, Bambino Gesù Children’s Hospital, IRCCS, Rome, Italy; ^2^ Gastroenterology and Nutrition Unit, Bambino Gesù Children’s Hospital, IRCCS, Rome, Italy

**Keywords:** congenital biliary dilatations, pancreatitis, endoscopic retrograde cholangiopancreatography, pancreatobiliary maljunction, pediatrics

## Abstract

**Background:**

Congenital biliary dilatation (CBD) is a congenital malformation of the main biliary tract usually associated with the pancreatobiliary maljunction (PBM), determining stone formation, cholangitis, pancreatitis, and cholangiocarcinoma. The role of endoscopic retrograde cholangiopancreatography (ERCP) in treatment and diagnosis has not been established yet. Therefore, the aim of our study is to define the actual role of ERCP in children with CBD.

**Methods:**

A retrospective review of consecutive patients with congenital biliary dilatation undergoing preoperative ERCP and subsequent surgical treatment at our pediatric tertiary referral center (Endoscopy and Digestive Surgery, Bambino Gesù Children's Hospital, IRCCS, Rome, Italy) was performed between 2012 and 2023.

**Results:**

A total of 31 patients were included in the present study. Preoperative ERCP detected a PBM in 28 patients (90%). According to Todani's classification, 2 patients (6.5%) had choledochal cyst (CC) type IV, and 29 patients (93.5%) were diagnosed with CC type I. In 18 (58%) patients, ERCP was performed for treating acute pancreatitis. Sphincterotomy could be performed in 23 of 31 (74%) patients. Patients who did not undergo sphincterotomy had a higher number of acute episodes while awaiting surgery.

**Conclusions:**

The present study is supportive of an essential role of ERCP in the diagnostic and preoperative management of children with CBD with acute presentation or inconclusive magnetic resonance cholangiopancreatography findings.

## 1 Introduction

Congenital biliary dilatation (CBD) refers to congenital malformations of the biliary tree characterized by an abnormal dilatation of the extrahepatic and/or intrahepatic bile ducts (IHBDs) without evident obstruction. These malformations are frequently associated with pancreatobiliary maljunctions (PBMs), an abnormal arrangement of the ductal system in which the pancreatic and biliary ducts join outside the sphincter of Oddi. This anomaly facilitates a double reflux mechanism that is believed to contribute to cystic dilatations. PBMs are classified by the Japanese Study Group into four types: (A) stenotic type, (B) non-stenotic type, (C) dilated channel type, and (D) complex type. CBDs are most commonly categorized using Todani's classification, although its subclassifications are sometimes difficult to assess (1, 2).

Surgical resection of all affected biliary tracts is the standard treatment for CBD due to the risk of cancer development in the affected tracts. Roux-en-Y hepaticojejunostomy is the gold standard surgical technique, yielding favorable outcomes, although long-term follow-up is advised (3–6). CBDs show a female preponderance, with an incidence of 1 in 150,000 live births in Western countries and up to 1 in 1,000 in East Asia. If left untreated, CBDs can lead to complications such as cholangitis, cholelithiasis, pancreatitis, and cholangiocarcinoma (7).

The clinical presentation varies with age. In infants, Type A PBM often presents as cystic dilatation, frequently detected prenatally. Older children may present with obstructive jaundice, acholic stools, or recurrent abdominal pain, although the classic triad of pain, jaundice, and palpable mass occurs in only 15% of cases. The majority of clinical data on pediatric PBM come from Asia, where the incidence is higher, highlighting potential regional differences (8).

Diagnosis typically involves abdominal ultrasound (US) and magnetic resonance cholangiopancreatography (MRCP), the latter being the gold standard non-invasive method despite variable sensitivity (3, 7, 9). Endoscopic retrograde cholangiopancreatography (ERCP) is occasionally employed for diagnosis and treatment due to its high success rates and reduced perioperative morbidity (10, 11). However, its use in children is limited by the invasive nature of the procedure and the risk of post-ERCP pancreatitis (PEP) (9).

This study aims to better define the role of ERCP in the diagnosis and management of pediatric CBD.

## 2 Methods

A single-institution retrospective long-term analysis of consecutive patients with CBD who underwent preoperative ERCP and subsequent resection of the biliary tract with Roux-en-Y hepaticojejunostomy was conducted at our pediatric tertiary referral center (Endoscopy and Digestive Surgery, Bambino Gesù Children's Hospital, IRCCS, Rome, Italy) between 2012 and 2023. Inclusion criteria were age <18 years.

Follow-up consisted of an annual or biannual outpatient visit with abdominal ultrasound and blood tests. PBMs were diagnosed following the classification of the Committee on Diagnostic Criteria of the Japanese Study Group. Patients underwent either US and ERCP or US, MRCP, and definitive ERCP for confirmation. The final diagnosis was made upon intraoperative findings and pathology. Sphincterotomy was performed when biliary lavage alone was not effective in removing the obstruction.

The primary outcome was to define the true role of ERCP in children with CBD. Clinical, endoscopic, radiological, and surgical information and data of the patients were collected and revised, along with those related to their follow-up and outcomes. Acute pancreatitis (AP) and acute recurrent pancreatitis (ARP) were defined according to the criteria established by the INSPPIRE consortium (12). For patients who underwent ERCP due to an acute pathology, the procedure was performed within 7 days following the diagnosis of AP. PEP was diagnosed in patients who experienced new or increased abdominal pain and increases in amylase or lipase levels to more than three times the upper limit of normal on the day after the procedure and no later than 2 weeks after the procedure. Severity was classified and reported as mild, moderate, severe, or fatal according to the 2010 American Society for Gastrointestinal Endoscopy lexicon for adverse events (13).

The sample size was determined by the total number of patients who underwent ERCP and subsequent biliary tract resection with Roux-en-Y hepaticojejunostomy at our center during the study period. Categorical variables were reported as absolute and relative frequencies when appropriate. For continuous variables, a Kolmogorov–Smirnov test for normality was performed. Continuous variables with normal distribution were reported as mean and standard deviation, and variables with non-normal distribution were reported as median and range.

Groups were compared using Fisher’s exact test for categorical variables. For non-normal distribution variables, differences between groups were established using a non-parametric Mann–Whitney *U* test. For normal distribution variables, differences between groups were established using Student’s t-test. All *p*-values were two-sided, and a value <0.05 was considered significant.

The present study received authorization for publication from the scientific board in the authors’ institution. Written informed consent was obtained from the minor(s)’ legal guardian/next of kin for the publication of any potentially identifiable images or data included in this article.

### 2.1 Endoscopic technique

A standard (Olympus Europe tjfQ190V) or a slim (Pentax Medical ED32-i10) duodenoscope was alternately employed, depending on patient's weight, the presence of associated altered anatomy (e.g., annular pancreas), and operator experience. Both duodenoscopes have a disposable distal attachment to minimize the risk of cross-contamination. A two-way Cotton's cannulotome or an ultrathin sphincterotome (Cook Medical) was employed, depending on the diameter of the major papilla. Depending on the chosen sphincterotome, a 0.32 or 0.18 mm Terumo guidewire was supported for the biliary cannulation.

## 3 Results

A total of 31 patients were included in the study (Table 1), of whom 74% were female (M:F 8:23). No patient had a history of prenatal diagnosis. Of the 31 patients, 18 (58%) patients presented with at least one episode of acute pancreatitis, 12 (38.7%) had a history of abdominal pain with evidence of acute biliary pathology, and one patient had recurrent abdominal pain associated with jaundice and hypocolic stools.

**Table 1:** Study population.

	Patients (*n*: 31)
Mean age at surgery (years)	6.9 (SD 5.2)
Median weight at surgery (kg)	18 (range 12–60)
Mean surgical time (min)	289 (SD 82)
Comorbidities (*n*)	
Down syndrome	2
Annular pancreas	3
Duodenal atresia	2
Presentation (*n*)	
Recurrent abdominal pain	12
Pancreatitis	18
Jaundice	1
Other	0
Choledochal cyst type (*n*)	
I	29
II	0
III	0
IV	2
V	0
Pancreatobiliary maljunction (*n*)	
Type A	10
Type B	8
Type C	5
Type D	6
No PBM	2
Diagnosis (*n*)	
US + ERCP	20
US + MRCP+ ERCP	11

US, abdominal ultrasound; ERCP, endoscopic retrograde cholangiopancreatography; MRCP, magnetic resonance cholangiopancreatography.

A total of four patients had associated comorbidities, two had a history of duodenal atresia, three had associated annular pancreas, and two had Down syndrome. The mean age at surgery was 6.9 years (SD 5.2). The mean weight at surgery was 24.2 kg (median 18, range 12–60 kg).

**Figure 1:**
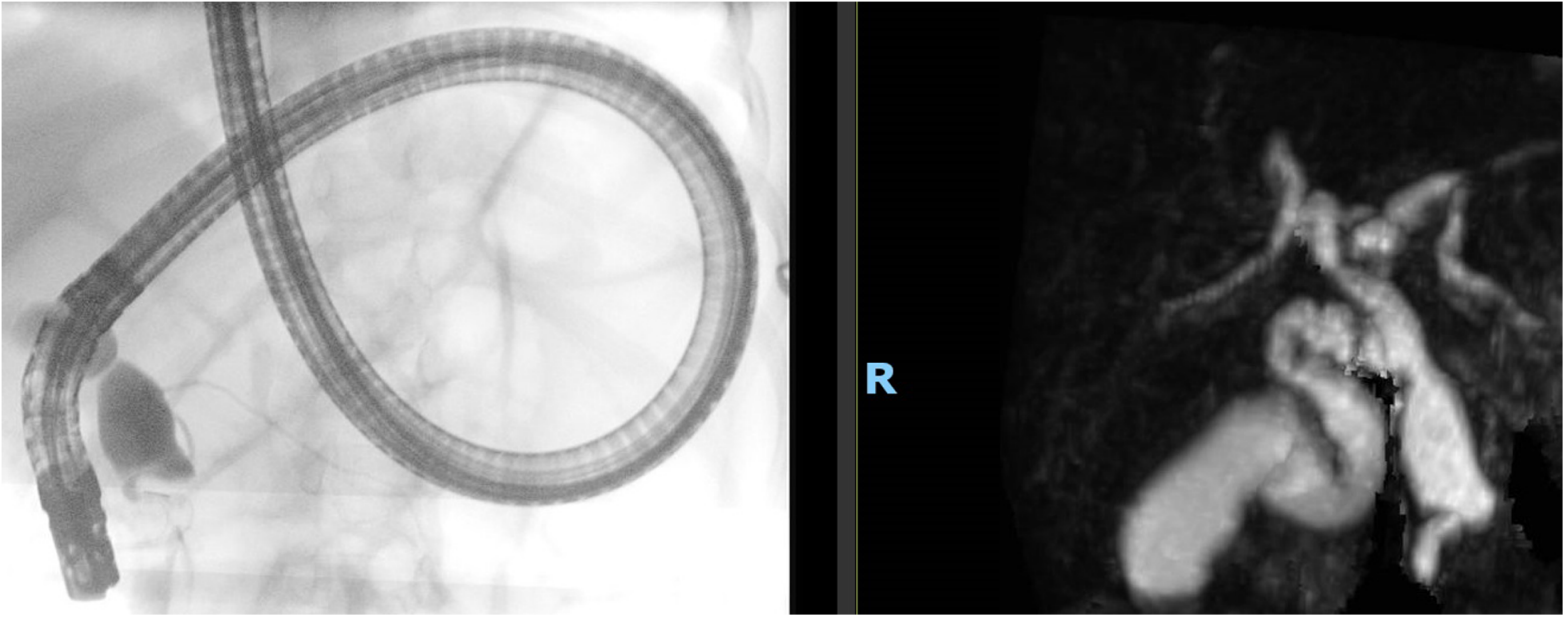
ERCP compared with MRCP.

All patients underwent ERCP (Figure 1). In 18 out of 31 (58%) patients, ERCP was performed for acute pancreatitis; in 12 out of 18 (66.7%) patients, there was evidence of choledocholithiasis; in 6 out of 18 (33.3%) cases, the procedure was performed for acute recurrent pancreatitis; and in the remaining patients, ERCP was performed due to inconclusive findings on MRCP. After patients regained stability, a standard sphincterotomy was successfully performed in 23 out of 31 (74.2%) patients. In the remaining 8 (25.8%) patients, sphincterotomy was not performed because biliary lavage produced satisfactory results (Table 2).

**Table 2:** Endoscopic results.

	Sphincterotomy (*n*: 23)	No sphincterotomy (*n*: 8)	*p* (<0.05)
Mean weight at ERCP (kg)	26.1 (SD 17.9)	18.6 (SD 10.18)	0.13
Visualization (%)			
Pancreatic duct	91.3%	87.5%	1
IHBD	52.1%	17.3%	1
Time to surgery (months)	3.8 (SD 0.5)	3.95 (SD 0.9)	0.35
Symptoms to surgery (*n*)	1	3	0.04

We observed that patients who did not receive sphincterotomy had a higher number of Emergency Department visits while awaiting surgery because of symptoms (abdominal pain, biliary colic, or pancreatitis) compared with those who received sphincterotomy. Pancreatobiliary maljunction was present in 28 out of 31 patients (90%). Type A PBM was recognized in 10 patients (32%), who were younger than those with other types of PBMs (Table 3).

**Table 3:** Clinical presentation of different PBMs.

	Type A (*n*: 10)	Type B (*n*: 8)	Type C (*n*: 5)	Type D (*n*: 6)	*p* (<0.05)
Mean age at presentation (years)	3.6 (SD 1.6)	10.3 (SD 6.7)	8.5 (SD 3.6)	6.9 (SD 3.6)	<0.05
Clinical presentation					
Pancreatitis	6	5	5	4	1
Abdominal pain	3	3	0	2	1
Jaundice	1	0	0	0	1

Clinical presentations were not statistically different between PBM subtypes, although a presentation with an episode of pancreatitis was common in our series (54%). A total of 29 out of 31 patients were diagnosed with CC type I (Todani classification) and 2 patients had CC type IV. The pancreatic duct (PD) was seen in 90.3% of patients, and IHBDs were seen in 64.5% of patients. The group that did not receive sphincterotomy had a lower rate of PD and IHBD visualization compared with the non-sphincterotomy group, but the difference between the groups was not statistically significant.

No intraoperative ERCP-related complications were noted in the patients. We report the case of one patient with mild PEP that occurred after sphincterotomy, which was treated conservatively with complete resolution. All patients underwent open resection of the biliary tract with Roux-en-Y hepaticojejunostomy. During the procedure, intraoperative cholangiography was performed in 7 out of 31 patients (22.5%), 4 of whom had PBM type B and the remaining 3 had PBM type D. In these patients, intraoperative cholangiography was performed to identify the correct level of resection.

No intraoperative complications occurred in the patients. The mean surgical time was 283 min (SD 82.6, median 269 ± 48.31). Two out of 31 patients were lost to follow-up. For the remaining patients, the mean follow-up time was 4.34 years (median 4.94 ± 0.92, range 0.23–10.5).

Four patients had complications (early, late, 1:3): a single early postsurgical leak was treated conservatively; late complications included one patient with recurrent pancreatitis on the choledochal stump and one patient with multiple malformations and subsequent surgery (duodenojejunostomy for duodenal atresia) who developed blind loop syndrome. These patients required a new surgical procedure.

In one patient, there was a stenosis of the biliary digestive anastomosis, which was treated with percutaneous dilatations. The last two patients mentioned had a history of neonatal surgery for other malformations that occurred prior to choledochal cyst resection (Table 4). All other patients were asymptomatic with stable blood examination and abdominal ultrasound results.

**Table 4:** Follow-up results.

	Sphincterotomy (*n*: 23)	No sphincterotomy (*n*: 8)	*p* (<0.05)
Early complications (*n*)	1	0	
Biliodigestive anastomotic leak			1.00
Late complications (*n*)			
Long choledocic stump	1	0	1.00
Blind loop syndromes	0	1	1.00
Biliodigestive anastomotic stenosis	0	1	1.00
Mean follow-up (years)	4.2 (SD 0.62)	4.3 (SD 1.1)	0.42

## 4 Discussion

In the preceding paragraphs, we described our ERCP-based management of pediatric patients with choledochal cysts presenting with acute pancreatitis or unclear findings on MRCP. The high efficacy and non-invasiveness of MRCP have significantly altered the diagnostic approach for suspected pediatric CBD. MRCP is confirmed to be a non-invasive study with a high detection rate of biliary dilatation (3, 7, 9). However, it has some limitations. MRCP requires sedation in non-cooperative children or infants, and even in cooperative patients, movement during the examination or complex junctions can affect the quality of the images, especially when the common channel is short.

In some patients, a definitive diagnosis of PBM and CBD necessitates direct cholangiography such as ERCP (9). Preoperative imaging is crucial for proper surgical planning in CBD management. Incomplete resection of the bile duct carries a long-term risk of cancer developing on the residual stump, underscoring the importance of obtaining the best visualization of the bile ducts and their junction. In our series, the rate of CC visualization was 100%, with maljunction identified in 90% of cases.

Clinical presentation with acute pancreatitis is observed in patients with PBM and choledochal cysts, with frequency rates ranging from 7.3% to 31%, and even higher rates recorded in some reports (7, 8, 14). We observed that patients in our series tended to be older. The rarity of PBM may lead to diagnostic delays in some patients, as recurrent biliary colic is often overlooked until a more severe presentation prompts an emergency department visit.

Biliary plugs are common in PBM. These lithostatic plugs tend to form due to a reflux mechanism associated with the maljunction. These stones are fragile and usually resolve spontaneously, causing transient symptoms that can generally be managed with conservative therapy (7). However, the presence of these plugs can lead to pancreatitis. Acute pancreatitis and evidence of acute biliary pathology without a known diagnosis of PBM and CBD warranted ERCP in 58% of the patients in our series. ERCP was both diagnostic and therapeutic in these patients, relieving the obstruction and confirming the diagnosis of CBD.

Furthermore, we observed that preoperative ERCP with sphincterotomy appeared to reduce symptoms in these patients, as this group had fewer emergency department visits while awaiting surgery compared with the group that received ERCP without sphincterotomy.

The main concern about using ERCP in pediatric patients is the risk of PEP. In pediatric patients, the risks associated with ERCP are similar to those in adults, including acute pancreatitis, hemorrhage, infection, and gastro-intestinal (GI) tract perforation (10, 15–17). Reported rates of postprocedural pancreatitis in pediatric patients vary widely, ranging from 2.5% to 10.9% (11, 18, 19). The risk of postprocedural pancreatitis is one of the most significant limitations of ERCP. A previous series reported by Iqbal et al. indicated a low rate of incidence of PEP (2.5%), with higher risks associated with therapeutic ERCP (11). However, a recent large series reported a prevalence rate of 12.8% (20). This discrepancy in reported rates may be partially explained by higher rates of pancreatic indications and higher ASGE complexity scores (21) in comparison with those in the previously reported literature. In addition, the lack of a single definition for pediatric PEP may influence the reported rates.

Another concern is that the use of ERCP in pediatric patients with AP is potentially risky, as the procedure may exacerbate the condition and lead to further complications. A recent multicentric prospective study by Trocchia et al. on the use of ERCP in pediatric patients presenting with acute pancreatitis found no significant difference in adverse event profiles between patients with and without AP, with a lower rate of complications (4.1% in AP cases vs. 10.6% in non-AP cases). Importantly, the rate of incidence of PEP was low in patients with AP, with only 1.6% of patients developing PEP, compared with a higher rate of incidence in non-AP cases (10.6%). This suggests that ERCP, when appropriately indicated and performed by experienced endoscopists, does not necessarily worsen the condition of acute pancreatitis (22).

Our study has some limitations, including its retrospective monocentric design and small sample size. A multicentric study may be advisable to confirm the effectiveness of preoperative ERCP in patients with CBD. Another limitation is that the procedure requires advanced endoscopic skills and should be performed in a referral pediatric center.

## 5 Conclusions

This study supports the essential role of ERCP in the diagnostic and preoperative management of children with CBD presenting acutely or those with inconclusive MRCP findings. When feasible, sphincterotomy reduces the number of readmissions in patients awaiting surgery.

## Data Availability

The raw data supporting the conclusions of this article will be made available by the authors without undue reservation.
